# P-2341. Was Viral Interference Responsible for the Unusual 2021-22 Influenza Season?

**DOI:** 10.1093/ofid/ofae631.2493

**Published:** 2025-01-29

**Authors:** Mary G Krauland, Mark S Roberts

**Affiliations:** University of Pittsburgh School of Public Health, Pittsburgh, Pennsylvania; University of Pittsburgh School of Public Health, Pittsburgh, Pennsylvania

## Abstract

**Background:**

Viral interference is a short-term, non-specific immunity that limits infection by a second virus when a person has been infected by a first virus. Epidemiological and biological data supports the existence of viral interference, most likely caused by interferon production in response to an infection. The 2021-22 influenza season in the US displayed a highly unusual pattern in which a single influenza variant displaying a bimodal pattern, with a fall rise in transmission followed by a winter decrease and subsequent increase. The addition of a refractory period to a simulation of co-circulation of an Omicron-variant COVID-19 like virus and an H3N2-like influenza virus produced similar transmission dynamics to that observed in the 2021-22 influenza season.**Figure 1.** Covid-19 Omicron variant and influenza cases in the US population and in the simulation.

**Figure:**
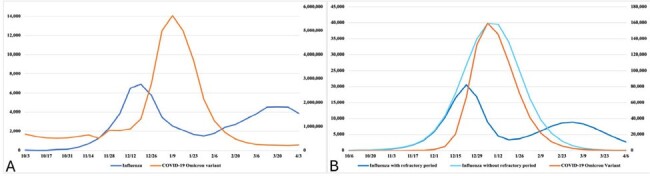
A: Reported Covid-19 Omicron variant and influenza cases in US in the 2021-22 season. B: Simulated COVID-19 and influenza cases with and without refractory period with varied prior immunity to COVID-19. Left Y-axis, influenza cases; right Y-axis, Covid-19 cases.

**Methods:**

We used the Agent-based modeling platform FRED (Framework for Reproducing Epidemiological Dynamics), with inclusion of a model of Omicron-variant COVID-19 like virus and an H3N2-like influenza virus.

**Results:**

In a simulation including a 7-day refractory period, the introduction of an Omicron-like variant in December of the season after influenza transmission was already widespread caused influenza transmission to subside while the Omicron-like variant was circulating at high rates. Influenza still circulated at lower levels and rebounded after the Omicron-like variant surge decreased since there was no cross-immunity between the 2 viruses and a large pool of influenza susceptible agents remained. The epidemic curve from this simulation resembled in shape the epidemic curve of H3N2 influenza in 2021-22 (Figure).

This effect was not present in similar simulations without a refractory period and was similar over the values of prior population immunity included in the simulation set.

**Conclusion:**

Viral interference caused by widespread COVID-19 Omicron variant transmission is a plausible explanation for the unusual influenza infection pattern seen in the 2021-22 season. It is likely that viral interference is often influential in the interactions of a number of respiratory pathogens and should therefore be included in simulations that include multiple co-circulating respiratory pathogens.

**Disclosures:**

All Authors: No reported disclosures

